# Exploring influences of health and wellbeing in Sydney’s apartment living: A qualitative study of residents’ perceptions

**DOI:** 10.1371/journal.pone.0329879

**Published:** 2025-08-06

**Authors:** Tamara Al-Obaidi, Jason Prior, Erica McIntyre

**Affiliations:** Institute for Sustainable Futures, University of Technology Sydney, Sydney, New South Wales, Australia; Catholic University of the Sacred Heart: Universita Cattolica del Sacro Cuore, ITALY

## Abstract

In Australia, and internationally, a shift is occurring towards high-density apartment living with initiatives and research showing an increased interest in the relations between health, wellbeing and apartment buildings. This study explores the complex associations between residents’ perceptions of their health and wellbeing and the apartment buildings where they live within the context of Sydney, Australia, as the case study. It challenges the fragmented approach previously used to study healthy apartment living and their underlying assumptions that do not account for a coupled human-environment systems view of health and wellbeing concerning apartment living. Qualitative research was used, which included in-depth, semi-structured interviews with 17 residents living in different apartment buildings, supplemented by fieldwork and narrated photographs. Using a structured iterative thematic analysis process, 20 areas of health and wellbeing influence (themes) were identified and further categorised using synthetic thinking into diverse, context-dependent, multilevel, and pervading influences. The findings from this exploratory study suggest a complex view of health and wellbeing by residents of apartment buildings and provide novel and important insights that have not been previously reported in such breadth.

## Introduction

Creating healthy environments is a human right, recently declared the United Nations in a historic move [1]. Also, people spend most of their time living in buildings, the majority of which are in the form of homes [2]; therefore, understanding how residential buildings influence health and wellbeing is vital. Built environments, including residential buildings, can influence and impact our physical and mental health through complex factors acting alone or together. Such factors may be biological (e.g., mould and dust), physical (e.g., building ventilation and thermal comfort), social (e.g., loneliness and social status), personal (e.g., gender and individual health status) or others (e.g., location and ownership) [3, Chapter 3]. Moreover, many societal and environmental challenges and changes are occurring that are complex and systemic in the nature of their factors, relations, and impacts. For example, the world recently grappled with a once in a 100-year health crisis—the COVID-19 pandemic. Researchers believe COVID-19 hotspots manifested mainly in cities, purporting that the way cities, neighbourhoods, and dwellings are planned has historically contributed to disease and public health challenges [4].

In addition to infectious diseases and their influence, urban health faces other complex challenges, including accommodating different demographics, widening inequities in health and risk distribution, ageing populations, chronic and non-chronic diseases, changes in lifestyle, demography and social organisations, and accelerating urban vulnerabilities from environmental change [5–8]. Humans are also altering fundamental earth processes and many aspects of the earth’s natural systems and resources [9]. Urbanisation and its impacts through urban development are considered one of the major pressures on our ecosystems, the ecosystem services they provide, and our planet. These pressures are causing direct and indirect health impacts with an urgent need to mitigate and adapt our living and built environments [10–12]. All these challenges show a complex mix of existing and future factors that can, directly and indirectly, influence health and wellbeing through apartment living. Crucially, they reinforce the need to look at influences across different systems instead of addressing them separately because such factors cause accumulated health effects at the housing level over time [13].

On buildings, recent research, systematic reviews and industry-led initiatives have demonstrated a new level of interest in understanding the complex health and wellbeing factors within the building sector [14–17]. In a recent review, Carmichael et al. concluded that buildings are complex, and this calls for a systems approach to consider the impacts of multiple risk factors on health in research [14]. Turcu et al.’s [17] umbrella review that mapped health evidence at different spatial scales, including buildings in relation to housing, showed a research focus on the neighbourhood compared to other spatial scales and a limited discussion of planetary health outcomes. Overall, these studies indicate that the complex nature of health requires systems thinking and a planetary health approach to address the coupled human-environment relationships that coalesce at apartment buildings.

Considering this literature, we perceive health and wellbeing concerning apartment living as a complex system with various intersecting factors that requires an ecological systems lens to explore health and wellbeing influences. Increasingly, these influences are recognised as being part of complex systems, where health and wellbeing emerge from interactions between multiple factors operating at various levels (e.g., cellular, molecular, individual, population and societal) and scales (e.g., cities, neighbourhoods, and buildings) of biological, power, and spatial organisation [6,18]. Health is viewed as a complex phenomenon, an emergent property that self-evolves in a dynamic, multilevel way across the life course [19–22]. In addition, humans cohabit a dynamic complex planet with other species [19,23]. This brings an ecological perspective to health that shifts the focus to interdependencies between individuals, groups of people, and their environment, where each living level becomes a function of complex systems [24,25]. Therefore, applying a coupled human-environment systems thinking approach to studying influences of health and wellbeing at apartment buildings is necessary to understand this complexity.

Despite acknowledging the multiple influences of health concerning apartment living in Sydney in previous studies; there is still a lack of empirical research that considers residents’ associations of health and wellbeing using a coupled human-environment systems perspective, as argued by the authors of this article elsewhere [26]. Previous research mostly considers factors separately, deals with specific subject matters (e.g., design factors), or targets specific resident groups (e.g., families with children) [27–33]. In addition, current challenges and emerging issues, including climate change and pandemics, are either not being dealt with together or only being addressed as impacts arise. Studies using complex systems and ecological thinking have yet to be undertaken in Australia to understand the nature and type of health and wellbeing influences with a focus on apartment living. This study uses a coupled human-environment systems perspective to explore what influences health and wellbeing by investigating residents’ views. It proposes another approach to understanding health and wellbeing influences concerning apartment buildings that complements existing studies in its use of holism and systems thinking.

In particular, the paper provides insights into the influences of healthy apartment living from the perspectives of residents who live in high-density apartment buildings using the city of Sydney as a case study. Specifically, the study aims to explore: 1) the nature of influences that residents of apartment buildings in Sydney associate with health and wellbeing; and 2) the type of influences that residents of apartment buildings in Sydney associate with health and wellbeing.

We explore these aims through qualitative methods, using primarily semi-structured interviews. Through this empirical research, we contribute to knowledge by illuminating unexplored influences of health and wellbeing together using a coupled human-environment systems approach for the first time. We also inform future urban health research and practice by mapping influences of health and wellbeing holistically and systemically.

## Methods

We used an interpretive exploration of residents’ perceptions and experiences to understand the influences of health and wellbeing regarding apartment living. The interpretive exploratory form of inquiry helped reveal what and how meanings were embodied in the language and descriptions of participants through a process of interpretation or understanding [34]. The data collection and analysis methods are reported below, with additional detail in the supplementary files.

### Research context

This study explored views from residents of high-density apartment buildings of four storeys and above. To explore the research questions, one of the largest cities in Australia by population (Sydney) in the State of New South Wales (NSW) was chosen as the study site. High-rise and higher-density apartment buildings have become the norm in Australia, promoted by an urban consolidation agenda in major cities. As a result, the number and proportion of residents living in apartments is increasing and set to continue. According to the ABS 2016 census data, the highest proportion of apartments as a portion of all occupied dwellings was in the state of NSW (at 21%), with 87% of apartments located in NSW’s capital city (Sydney) [35]. NSW is also considered one of the biggest drivers of change in terms of the number of dwelling commencements for medium-rise (4–8 storeys), high-rise (9–19 Storeys), and super high-rise (20 or more storeys) apartment buildings in Australia [36].

The findings reported in this paper are part of a doctorate research study conducted entirely by the first author (TA) to explore residents’ views of health and wellbeing concerning high-density apartment buildings in Sydney. Co-authors (JP) and (EM) supervised the study, including the data analysis and findings reported in this paper.

### Study design

This study adopted a reflexive qualitative design consisting of field visits to select suitable apartment buildings across Sydney and semi-structured interviews and narrated photographs with residents of those apartment buildings to answer the following research question: what influences health and wellbeing concerning apartment buildings in Sydney from their perspectives? The study’s methods and findings follow the COREQ guidelines for qualitative research [37].

### Field visits and participants’ recruitment

The holistic nature of the research meant we sought participants from a wide range of backgrounds, apartment buildings, and suburbs. This is compatible with the researchers of this paper, who have cross-disciplinary research and professional backgrounds in different fields, including environmental and public health, planetary health, urban planning and design, and sustainable development.

A combined purposeful sampling strategy was used to recruit participants [38]. This process involved randomly selecting resident cases rich in information and of maximum variation to illuminate the complex views of health and wellbeing in the context of apartment buildings in Sydney. Participants were selected following extensive field visits that took place several times from March 2022 until August 2022 to choose buildings that fit the criteria described in the supplementary S1 File. Buildings were selected based on their geographic locations across Sydney, the walking distance between them to nearest transport hubs, their total storey level, and year of construction. Participants were recruited across Sydney from three geographic locations with different environments: buildings near the coast, buildings near the Parramatta River (a central location in the broader city of Sydney), and buildings inland in the far west of Sydney. This building selection represented the existing apartment stock distribution and climatic variations across the city. Minimum criteria were used to include participants based on age, household representation, tenure status, language, and being from each of Sydney’s most dominant high-density building submarket groups. The selection criteria of participants are described in S2 File.

Participants were invited by letters or handouts with information about the purpose of the study, which was to understand what a healthy apartment building meant to them. Invitation letters (1,600) were delivered to letterboxes or handed out across 45 buildings in 18 Sydney suburbs. Interested participants responded by email or by calling a mobile number. Ten residents were recruited to the study using this strategy. As the data collection unfolded and with time, we complemented this sensitising concept sampling with convenient and snowball sampling [38]—both commonly used in qualitative research to understand residents’ perceptions of urban health [39]. Therefore, additional recruitment methods, including word of mouth, a recruitment flier, and social media advertisement, were used. This resulted in seven more recruits to the study, bringing the total number of participants to 17. The sample size was deemed appropriate for a heterogeneous qualitative sampling study. In general, there are no rules for sample sizes, with various recommendations including reaching saturation—when little new information comes during data collection [38–40]. However, data saturation is not considered a concern in qualitative reflexive studies [41–43]. Answering the research questions took precedence over saturation where priority was given to the quality and relevance of participants’ information over quantity of participants included [41]. The characteristics of residents recruited are shown in the supplementary S3 Table and further explained under subsection participant characteristics. Ethical approval was obtained from the University of Technology Sydney (ETH21–6605) and then amended (ETH22–7156) for the additional recruitment methods.

### Data collection

One-on-one interviews with residents of apartment buildings (n = 17) were the primary data collection method, supplemented with narrated photographs. These qualitative methods enabled our understanding of how residents perceived their surroundings, including factors considered harmful or supportive to health [39]. All interviews were conducted by the first author (TA) online to ensure participants’ safety during the COVID-19 pandemic between April 2022 and August 2022. Participants were also offered face-to-face interviews if they preferred this arrangement. All interviews followed a semi-structured interview guide with questions and probes developed under the supervision of (JP) and (EM).

**Fig 1:**
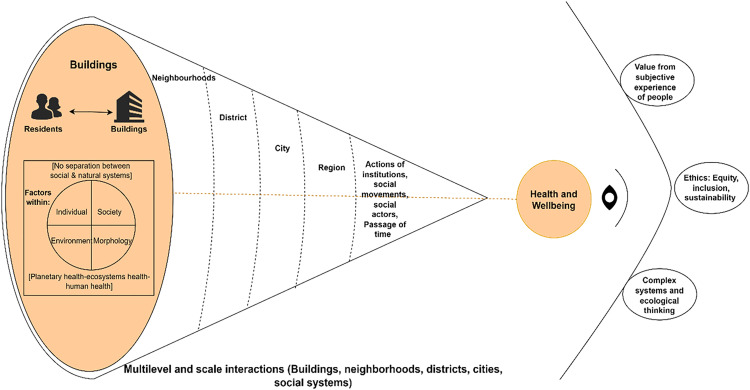
Conceptual framework to understand influences of health and wellbeing concerning apartment buildings within local context.

The interview guide had ‘softly’ defined concepts and adaptable questions, which were modified to be consistent with the iterative nature of the qualitative study. These concepts were derived from a social-ecological framework developed by the first author (TA) for the interpretation of the research findings. It captures the interaction between residents, apartment buildings, health and wellbeing, and other influences using concepts derived from a literature review and understanding of available approaches to healthy urbanism. Behind the framework’s ideology is a relational complexity view of reality based on dynamic and open interactions between systems where multiple causes, whether quantifiable or not, are considered together. The framework conceptualises the main domains that can influence health and wellbeing concerning apartment living as operating at, and interacting within or between multiple levels and scales of social organisation. Fig 1 summarises the conceptual framework. The rationale behind the framework’s development is explained in the supplementary S4 File.

Incorporating complex systems and ecological assumptions assume nonlinearity, heterogeneity of components, holistic takes, and emphasises the interwoven nature between human and environmental interactions [26]. Accordingly, the complex systems and ecological perspective helped explore factors and concepts simultaneously. It also helped inform the design of the interview guide by reflecting the concepts of systems thinking, ecological public health, healthy urban development principles, and the main areas (domains) of health and wellbeing influences from previous models and frameworks as explained in supplementary S4 File. The interview guide included questions that explored influences on health and wellbeing within urban environments (e.g., social aspects, environmental stressors, equitable apartment buildings). Participants were asked “what makes an apartment building healthy” using individual, social, building aspects, sense of place, and environmental stressors, which were derived from a selection of domains informing the conceptual framework. Participants were also asked about equitable, inclusive, and sustainable apartment buildings corresponding with the underlying principles behind the study’s conceptual framework. Probing questions were used to elicit further discussions about the topics in question and to understand behaviours and actions undertaken by participants. The original interview guide with which the ethics application of the study was granted and its formulation rationale can be found in the supplementary S5 File. The interview guide was developed based on existing qualitative research design guidance books [38,40]. The guide was piloted with residents of high-density apartment buildings (first author’s friends) prior to commencing the interviews with participants in Sydney.

Each interview session had one interviewer and one participant. Only one participant out of 17 had prior interactions with the interviewer. All participants provided informed consent verbally or electronically and received a $30 AUD electronic grocery gift card for participating in the research. Verbal consent was obtained prior to beginning the interviews while written consent was provided either electronically or by post with participant signatures in accordance with the University’s ethics approval (ETH21¬6605). In both cases, consent was witnessed and documented by the first author (TA). Interviews were conducted using Microsoft Teams (version 1.7.00.15969), a secure, suitably encrypted program. The interviews were audio and video recorded and ranged between 45 minutes to two hours. After recordings, the interviews were transcribed verbatim using two professional transcription services and the interviewer (TA). In the case of third-party transcriptions, appropriate protections were put in place for data security. All interview transcripts were emailed to research participants for their review and verification following each interview. To maintain privacy, all participants were assigned pseudonyms.

In the interviews, participants were offered the option to complement their accounts with narrated photographs as an additional structured data collection technique [38,40]. Such techniques can reveal participants’ understanding of their surroundings, especially from the perspectives of those who find it easier to communicate and express their opinions in nonverbal ways [39,44]. This secondary method complemented the semi-structured interviews and ensured inclusivity among participants. Two out of the 17 participants provided photographs. Photographs were supplied by email following the interviews with simple written reflections. None of the photographs is presented to prevent accidental identification of participants’ residences.

### Data analysis

The first author (TA) used a thematic analysis approach to code the data, namely template analysis with reflexivity for a flexible and organic analytical process [42,45]. The thematic analysis process involved: data familiarisation, preliminary coding, clustering, producing an initial template, applying and developing the template, final interpretation, write up, and quality checks. This approach allowed for a structured data coding process while recognising the researcher’s subjectivity for this qualitative study [42]. Data from interviews and any narrations added by participants were coded together in two steps: manually, then again in NVivo (QSR International Pty Ltd, version 12, 2022) to ensure further data reflections. Analysis was a cyclical process where TA analysed the text, identified codes, and revised and grouped codes under relevant clusters. These clusters were then verified by continually adding to and revising the codes. The analysis was conducted several times (four rounds), creating a ‘template’ of the clustered codes (themes); therefore, data analysis was a continuous process from the beginning of data collection, where an initial template was created until the final thematic output (the final template). Data were coded inductively and deductively [46]. Initial coding remained close to the data (in-vivo coding); however, clustering of codes was influenced by the softly defined concepts of the analysis framework, and the professional expertise in environmental health and personal experience of TA consistent with the template analysis method and the creative process of theme development [42,45]. Quality checks were applied throughout the analysis process using multiple methods to ensure the research met the trustworthiness criteria of credibility, dependability, confirmability, transferability, and triangulation for qualitative research (see Supplementary S6 File). An audit trail was used throughout the template analysis coding cycle with detailed explanations, modifications, and rich reflexive memos recorded and actioned.

An interpretivist approach informed by systems thinking was applied by TA to elucidate the findings using the reflexive template analysis process and further thematic synthesis. Systems thinking draws attention to holistic views of entangled complex situations embedded in context yet still considered parts of larger entities ‘systems’ [38,47]. Applying a systems thinking lens enabled deeper insights into the data and brought to analysis: a holistic overview of the situation, a recognition of the importance of interactions and relationships between parts, a recognition of the multiple levels of systems, and accepting the divergent rationalities and purposes of participants [48]. Through thematic analysis, three tiers of themes were developed into codes, sub-themes, and overarching themes. Only the higher order overarching themes are described in the results section, followed by further synthesis of the 20 themes into four layers of complexity to understand influences systemically and wholistically [49]. This combined analysis and synthesis is essential to systems thinking and helped explain the holistic nature of health and wellbeing influences concerning apartment buildings in Sydney. The themes and the four layers of complexity are then discussed together in the discussion section. The thematic output of the study (the 20 themes, subthemes and factors) is published on a separate Figshare webpage [50].

## Results

### Participant characteristics

In total, 17 participants were interviewed with two of these providing additional narrated photographs. Participants came from across the coast, middle and west of Sydney. Their characteristics varied in every sociodemographic category, including age, work and/or study status, gender, household type, apartment building tenure, and income. Apartment buildings were located across different suburbs in Sydney and in different apartment storey levels of 2, 4, 5, 6, 7, 8, 10, and 13. Nine participants identified as male and eight as female. All participant characteristics are presented in the supplementary file S3 Table.

The differences in apartment building dwellers across the different suburbs imply a heterogeneous mix of typical occupants in Sydney, consistent with previous studies [51,52]. Participants’ profiles suggest that there could be other unrecognised submarket groups of apartment dwellers in Sydney, including same-sex couples, families with children born overseas with a weekly income above $1499 AUD, and single parents with dependent children below the age of 18. It also became evident during the recruitment stage that participants with social housing arrangements are another group occupying Sydney’s ‘undesignated’ apartment buildings. However, none were recruited in this study.

### Themes

The analysis resulted in 20 high-level overarching themes perceived by residents as influencing their health and wellbeing concerning high-density apartment living in Sydney. All 20 themes (areas of health and wellbeing influence) are listed and defined in the supplementary file S7 Table. The number of themes identified in this study makes presenting and discussing all themes impractical. Here, we have focused on the 10 themes that are surprising for their lack of prominence or consideration in previous research. The remaining themes add to previous research [27–33] by expanding on the scope of factors considered and described. These remaining themes represent other key areas of influence to participants’ health and wellbeing related to the apartment day-to-day use, an apartment buildings’ functionality, management and physical location, apartment building stressors, the needs of residents and their ability to socialise with others as well as the role of the surrounding area and neighbourhood of the apartment building. The themes are presented in the sequence of their discussion and grouping in the next discussion and implication section of this paper. Below, we present a selection of participant quotes (with pseudonyms) that reflect each theme with additional examples included in the supplementary S8 Table.

#### Theme – Living with nature sustainably.

Almost all participants spoke of the need to live and exist with nature in sustainable ways, including through the sustainable design and planning of apartment buildings.

Most participants provided detailed examples of factors related to sustainable design and planning of apartment buildings. For example, one participant commented in detail on the provision of solar power and electricity management in the building as two factors related to this theme:

*“The thing that I would like, the thing that I missed, because I had solar energy up in the mountains on my house, why can’t apartments get that together? Why can’t they organise people to put money together and put solar panels on the roof or something? It worries me a lot about the electricity that’s used in this building. Downstairs in the car park, for instance, there’s an alarm that’s been going. It’s a red flashing light and I’ve told the building manager about it and he’s sort of come and stopped the noise because it was making noise […]but the red flashing light’s still going. It’s been going for two months. I feel like going down there and smashing it and saying stop wasting electricity day and night. It’s not even doing anything, it’s apparently alarm about the sewer. I have to write a letter to the building manager, but those sort of things bother me a lot when there’s a waste of electricity. When you walk out the corridor, all the lights come on, but I suppose that’s convenient. Down the car park, when you drive your car and all these lights come on, or I just think, oh, the amount of electricity. It’s just does bother me quite a bit, so why can’t we get together to have solar panels and do better? That’s one thing that does frustrate me.”*

(Lux)

Another participant describes the measures that some participants undertook to ensure a way of living with nature sustainably:

*“Um, another thing about. Umm, like we’re both quite like, I suppose you’d call greenies. Not sort of over the top, but we, uh are interested in the electric car but this apartment block…Umm…Well, we had to push, well…I had to push to get the garage electrified so that you could have charging stations in the garage and that sort of thing. Fortunately I am on the Executive Committee So I could raise it there and we’ve now…Umm just finished installing the necessary stuff to have car charging, which is good. We’re, also the rest of the committee is also quite…Umm Climate conscious and we’ve actually done quite a lot to build a sort of green flavour into how the apartments run...”*

(Jon)

#### Theme – Powers of renters.

Some participants reflected on how much authority and control renters of apartment buildings have in Sydney. When reflecting on the constant move between different places, one participant mentioned several factors that spoke of this pattern, including the increase in rent, renters being at the mercy of landlords, and renters moving around:

*“…moving all the time is not ideal. Like ideally I would like to find a spot where I’m comfortable and happy and I’d like to commit to like a two or three year lease and just. Not have to worry about when I’m going to find the next apartment or, you know, doing this sort of thing every year or worrying about whether the landlord would increase the price of our rental on a whim. So it does feel like you’re quite at the mercy of landlords as a renter in Sydney.”*

(Lou)

In another example, the same participant talked about the influence of real estate agencies in managing apartment issues and how this factor is perceived to make a difference to the participant’s health and wellbeing through the agency’s authority and duty over controlling mould:

*“The mould is a big factor. Umm, like we told our real estate agents that we moved in and there was a mouldy ceiling and they were like, yeah, that happens like the ventilation within the building isn’t good. That’s like, wow, that’s not really an acceptable answer to that solution.”*

(Lou)

#### Theme – Apartment ownership cost.

Few participants who either owned or were previous owners of apartment buildings mentioned factors that spoke of the expense of owning apartment buildings in Sydney. One participant commented on the cost of apartment ownership:

*“…the way things are going is like it is ridiculous, so as much as we own like this apartment, it’s like it is disgusting the amount of money that you have to like fork out and we are, I would say we are very fortunate in terms of we both have good jobs like we didn’t have a kid back then so we are in a very, very, very privileged position, […]And again, it’s just that typical thing of like feeling more secure by having your own home, blah blah blah like and like for us again like you know, not putting money in savings accounts and you know this is a. This is a better investment for us if you like and so yeah, we are super fortunate.”*

(Ani)

While another participant talked about the high cost of repairs incurred by owners, especially in buildings with regular problems:

*“I think the other just small thing could be […] what they call it the building infrastructure…The breaking…You could be in somewhere where it’s got regular problems, breaking down, not to mention, if you’re an owner. And. There’s a big repair that’s needed to the whole building, and then you’ve gotta to pay some massive fee like I. In Brisbane, the one I owned, there was a big hail storm and it damaged a lot of the air conditioners so everyone is like a lucky tip, like everybody whose air conditioning thing got damaged, had to pay a huge thing.”*

(Steffan)

#### Theme – Co-existence with neighbours/residents.

Participants reported the importance of how residents exist, be, socialise and live alongside other neighbours and residents of the same apartment building. Few participants commented on the extent of interaction they prefer with their neighbours. In the case of one participant, her preference was to know her neighbours but be able to be left alone out of respect for their privacy:

*“…being able to know your neighbours, but also […] being able to be left alone, if that makes sense. So you know, you don’t have to interact, you’re not forced to interact with them. We don’t really…We moved in during COVID, so it was difficult to meet neighbours. We have met them. We don’t have a lot in common with them. They’re very nice and we’re obviously nice to them as well. But we don’t, we haven’t met them that way. But you know, one of the first things they said to us was like, if you need anything at all, you know, please, please, please don’t. Don’t. Yeah. Don’t feel afraid to just knock on our door.”*

(Ada)

Another factor that some participants spoke of was the social environment level in apartment buildings. While reflecting on the social environment in his building, one participant also mentioned other relevant factors under this theme, including the frequency of meeting neighbours in the building, the friendliness between neighbours, and socialising with neighbours:

*“You’re living in a community of 100 apartments, you don’t actually get to meet people. It’s not, it’s not a very social environment because there’s security on the front door…there is security on the lift only goes to your floor…Security on your apartment. There’s only two apartments on our floor. So and we’re friendly with the other people on our now floor, the other family, but don’t socialise with them. …yeah, you don’t. You don’t really. It’s not a sort of social arrangement, which perhaps it could do with a bit of…a bit of work, but…but yeah, so. …That’s not so good.”*

(Jon)

#### Theme – Emergent health conditions.

The way emergent health conditions, especially COVID-19 influenced residents’ lives in association with other factors was referenced by almost all participants in one way or another. While discussing COVID-19, one participant commented on the level of susceptibility to infectious agents, especially in common areas:

*“Yeah, I guess definitely with COVID having more people around, you’re more susceptible to things and sharing a lot more common spaces with people. Um. I guess you just be a little bit more cautious with it.”*

(Sandra)

Most participants also brought up the subject of COVID-19 while talking about factors that may influence health and wellbeing. For example, one participant mentioned the importance of having a balcony as a blessing during COVID-lockdowns:

*“I know that some apartments don’t have much of a balcony, but I think it, you know, if you can have one, it definitely helps because you know, in periods that you wouldn’t expect like lockdowns, they could be a real blessing for people in apartment buildings.”*

(Rence)

Similarly, for another participant, the COVID period appears to have brought more appreciation for other aspects, including the number of apartment windows, the amount of sunshine entering the apartment, and having access to the outside of one’s apartment:

*“[…] other physical elements that might include lots of sunlight and windows and being able to, especially over this COVID period, have as much of outside in, if that makes sense, without necessarily having to go outside.”*

(Valerie)

#### Theme – Extreme weather events.

Some participants spoke of global and local weather events considered extreme or unusual, which may have already or have the potential to affect them unexpectedly. One participant alarmingly put this theme into perspective. While discussing his issue with controlling mould and the lack of windows in his apartment, he mentioned the frequency of wet weather events in Sydney as an influencing factor, bringing attention to the importance of considering global and local developments:

*“I think mould is a big thing. That’s been a thing I’ve been experiencing in my place recently. That has been something that I’ve also been trying to figure out as well, because if you’re in a space that doesn’t have a window…I generally have to keep my balcony open all the time when I’m home, and if not, I put on the dehumidifier, otherwise – yeah, we’ve had a lot of wet weather these past few months as well, so I can see how that’s probably affected it as well, but yeah, I’m still trying to get to the bottom of that.”*

(Matt)

Another participant illustrated an awareness of climate change while reflecting on extreme weather events and the number of residents that can be affected by such events when she commented on the 2022 NSW floods that had just taken place before the interview:

*“You know the environment and where we’re going in the future of climate change and all of that sort of stuff, I think also something to factor in, like with the recent floodings like…Our carpark was OK, but I know other people… like the drainage issues in carpark […] it goes so far underground, I think ours is 6 floors underground. So you know there’s a lot of drainage within that, but if it’s more of a one-story thing like with the amount of water that we had coming in, they just weren’t prepared for it. And we saw a lot of people whose cars were damaged […]. So that wasn’t in our building, luckily, but yeah, it’s one of those things where that’s going to happen. Well, frequently the way that we’re going. So we do need to make changes and factor things like that in more extreme weather events and that… especially yeah, because when you think about it like apartments, a high density living, so you’re like if something happens, it’s going to affect way more people than it does in like a suburban housing area because you know, we’re 19 floors, I would say that I can’t really guess the numbers, but you know we have quite a lot of people and it’s not a huge block. So… So yeah, it’s it affects a lot more people in one space. Umm. And yeah, it won’t be good […] if you know we’re all trapped in here and we can’t get out or anything like that.”*

(Lou)

While talking about climate change and extreme weather events, this participant linked these factors with other themes by discussing the levels of the building car park, car park drainage, building storey number, and residents being trapped in the building.

The prominence of extreme event influences was greater with some participants compared to others, which may indicate underlying issues regarding certain apartment buildings. For example, apartment design may have influenced how apartment buildings performed during the COVID-19 pandemic and how extreme weather events were experienced.

#### Theme – Government vision, processes and actions.

Participants talked about the role of government in influencing how residents experience residential developments. Through their foresight, processes and actions, different tiers of government (whether local, state or federal governments) appear to influence health and wellbeing through apartment buildings. More specifically, participants reflected on planning vision, integrative planning, actions through regulations, acting on the environment, quality control, and the overarching city vision of Sydney.

One participant’s views on consolidating Sydney were concerned with the building height limitations set by his local government as an influencing factor. They linked these government limitations with the surrounding area and neighbourhood level by citing factors such as the number of suburb facilities, area unpleasantness, and the effectiveness of public transport:

*“You have all these green, lefty people that want immigration, want refugees, but don’t want sprawl, and yet they won’t consolidate urban areas and it just makes no sense. And we’re left with ugly areas like Mascot perhaps here as well, where there’s very low height limit. So it is the city of Sydney, really. And actually beyond that, I find the city of villages crap. So annoying, so annoying. It’s so wrong.* Umm*, it should be a really important regional hub, regional CBD, and somehow we’re in the city of Sydney here and it’s treated like a we…we’re not a village, it’s an urban inner urban suburb you know, we need consolidation. If we had that, I’m sure we’d be able to have more facilities. We don’t have a full-service supermarket. You know, and so I have to drive out to say, Broadway or Marrickville metro it would be much handier…where, when? When is it coming? Why can’t we? And if we had higher density then we would have more rationale for better public transport proper, you know MRT’s etcetera, underground, rail. I just don’t understand Sydney city. It’s very misguided, but that’s just my little opinion. Most people probably don’t wanna live in Hong Kong, whereas I do, so you know*.”

(George)

Similarly, but addressing different factors, the participant (Martin) commented on the limitations of government bailiwick, the planning for infrastructure, and the scaling of services provided in the suburb with developments, revealing his thoughts on integration during the planning of apartment building developments:

*“I remember saying to someone from the Council who was manning the stand that day, I said, “Look, you know, the trains are pretty busy.” This was six or seven years ago. “The trains are pretty busy around peak, and the buses are busy and infrequent. You’re about to add eight to ten thousand more people. Part of the design of the new areas is, as you know, would have learnt, there’s fewer and fewer car parks, and you’re not allowed on-street parking permits, so I don’t bring a car here. So, you’re bringing eight to ten thousand people, who you’re trying to incentivise not to have a car, but the trains and buses are at peak. What’s the plan there?” (Slight laugh) And the response was, “That’s not a Council issue. That’s a state government issue.” And that just blew my mind (slight laugh). Like…you’re responsible for how many people who live here; someone else is responsible to making sure they have access to regular public transport. And that, to me, has been a constant issue. So, every time there’s a block development being proposed, whether it’s this building’s Facebook, or the inner west, Erskineville Facebook, someone will start complaining. “It’s above ten storeys, it’s out of character,” all that sort of stuff. But very few people will do the maths around…I don’t care if it’s eight or ten, or 15: it’s X hundred more people. You know. They’re not scaling services in lockstep. So, that is an issue.”*

(Martin)

#### Theme – Thinking sustainably.

Participants spoke about the importance of thinking of apartment buildings in sustainable ways. Ways that ensure any apartment buildings benefit people and the environment and achieve ecological balance.

While reflecting on the relationship between our health and the natural environment through apartment living, one participant linked the process of rehabilitating contaminated land and its benefits to the natural environment:

*“…so sometimes making these developments is actually good for the environment because…If the developers hadn’t have wanted to make money by selling their apartments then this whole area would have just stayed as the toxic waste ground there it was.”*

(Steffan)

Another participant commented on the link between people and the environment by referencing the act of building on wetlands, leaving places for species, and leaving existing wetlands while developing apartment buildings:

*“Look, I think we can go the wrong way with this whole apartment side of things, because I mean this was built on wetlands, which there’s a lot of species that lived on these wetlands. Because I know there’s a vacant lot on the other side of me, and they’ve just had the frogs mating, the golden bell frogs, so there’s all these little frogs. They’re losing their habitat bit by bit, and there’s not really places for them to go anymore. But I do notice that the bird life is just amazing here. […] They seem to coexist here, too, and they planted a lot of trees, which I think’s fantastic. Like I said, we’ve got the wetlands, like the canal. It’s - you’ve got your ducks and stuff like that, so that’s creating an ecosystem within our immediate area. On the other side, they’ve just left those natural wetlands, which I’m really happy about. There’s a small like little - you’ve got a wooden walkway that goes all the way around the wetlands, and yeah, it’s just - they’re still keeping it, but at the same time, they’re taking it. Do you know what I mean? There was a lot of animals that would have been displaced by the whole building of these estates, and if they include them in their planning and infrastructure, I think that’s a very important thing.”*

(Trevor)

Through different ways and processes, this participant linked the health and survival of the natural environment with our health and wellbeing. By elevating his thinking to the planning and infrastructure level, the participant took this link between humans and the environment to a structural level, where he alludes to the ways apartment building developments and the associated infrastructure are planned and their sustainability when it comes to the health and survival of humans and the natural environment.

Interestingly, some participants viewed the incorporation of nature in apartment buildings as having an aesthetic purpose. For example, one of the photographs provided by participant (Ada) had the comment: *“Greenery is more for aesthetics than sustainability.”* She further commented:

*“…our building, but it also I think most buildings I’ve seen don’t put a lot into it other than aesthetics. So our pool area for example has like a bamboo. Uh, surroundings. And that’s mostly shielded from, like commuters going past on the train but also it looks, it looks nice. Like really, it’s very shady. And you know, when you, when you go down, hang down the pool, it looks very, I don’t know hotel like resort like… (Laughter)… they do also have, like a few plants here and there. Yeah, like around the sort of the entrance area. But like I said, that’s mostly aesthetic is actually not for wellbeing it’s picked for. It’s something that’s easy to grow and maintain.”*

(Ada)

#### Theme – Place belonging.

Participants reflected on the importance of feeling at home or belonging to where one lives while living in apartment buildings from a social sense rather than a physical sense. In the case of one participant, her description of sense of belonging and the way it is influenced by and transcends through the apartment building, neighbourhood, and people around show the ‘invisible’ role of place in influencing participants’ health and wellbeing through several factors:

*“I mean originally the location, so again, here in Penrith has a stereotype. I grew up in Newtown, inner west Sydney. So, very much you sort of, all of Western Sydney, Penrith, Mount Druitt, St. Mary’s [has] very much a reputation around that, negative reputation around that. So first, coming out here, I thought, oh my gosh, I don’t know how I’m going to feel about walking to the shops or going anywhere. Being able to spend some time here and it really didn’t take me long. I really did find that sense of belonging in the area.”*

(Valerie)

Another participant commented on being from a religious group and being British immigrant in Australia and the extent of difficulty belonging, which allude to a complex, context-dependent relationship between place in a social sense, space (the physical location of apartment buildings in Sydney), and the influence over health and wellbeing:

*“So I’m. I moved to Australia about 10 years ago. My husband probably moved here about 15 years ago. We met only like so we met six years ago and because. Yeah, like I said, our immediate families aren’t in the country. […]. So we had two really good friends like two sets of friends. Like one yeah moved internationally, the one moved state so it’s as if international […] everyone’s having like kids, or they live in the suburbs and so it’s actually to feel like you’ve got a close kind of community is quite hard sometimes, like so we’re Muslim and we had Eid the other day and I. And I was saying (laughter), [inaudible segment] oh. Oh. Maybe we could do it next year […] both those two sets of friends that left were like are the two like Muslim kind of friends and families that we would like at least meet up on Eid and do something so those guys left both like a year or two years ago. So like our Eids have become like just the three of us like we’ll go to the mosque […] and will wander and like we’ll go to the cafe around the corner become a bit of a tradition […]”*

(Ani)

In the excerpt above we see the participant associating sense of belonging with other influencers, including the ability to celebrate religious occasions and being close to one’s social circle. During the interview, this participant also alluded to how difficult it is to make friends in Australia and the difficulty of belonging, which also seems to influence her sense of belonging.

#### Theme – Quality in building and infrastructure.

Participants discussed a range of factors that spoke to the importance of well-built apartment buildings and satisfactory and effective infrastructure alongside residential developments. One participant (Ryan) discussed several factors related to the quality of construction and living aspects of apartment buildings by linking quality with influencing factors that speak of ‘apartment day-to-day use’, and ‘building management’:

*“So, poor construction, as in, for soundproofing noise reduction. Again, poor construction.”*

(Ryan)

*“Some places I’ve had good building management and other places I’ve had bad building management where, as I said previously, where to get any change done or anything, you’ve got to band together and get a whole bunch of people involved in something, otherwise they just – yeah, they’re not really interested.”*

(Ryan)

In participant (Ryan)’s excerpts, quality was also linked with ‘government vision, processes and actions’.

Few participants also reflected on the quality and adequacy of public transport and area parking. For one participant, the inadequacy of area parking in Parramatta coupled with the lack of visitor parking in her building appears to influence her behaviour (e.g., having visitors over):

*“The other thing that I have found, and I knew it would actually be an issue for me, is, there’s no visitor parking, and Parramatta’s just terrible with its parking, so that’s, you know, that’s a difficulty if people are coming to see me, or I’ve got friends coming, or whatever”*

(Jane)

The affordability and limited parking options in (Jane)’s suburb, along with the lack of car parking spaces in her building, seem to have compounded and put the onerous on the participant and her guests to try to find alternative solutions.

### Further synthesis

Further analysis of the 20 themes showed four layers of complexity of health and wellbeing reported in participants’ data.

1. Diverse influences: health and wellbeing are influenced by a diverse web of factors. These influences emerge from residents, society, built environment, natural environment, stressors, and tenancy,
2. Context-dependent influences: residents’ views exposed unique and new influences of health and wellbeing, which are context dependent. Therefore, context influences health and wellbeing,
3. Multilevel influences: residents’ views demonstrated that health and wellbeing influences are not confined to the apartment building level, and
4. Pervading influences: residents’ views demonstrate the importance of having equity, sustainability, inclusivity, belonging and quality as overarching principles and necessities for healthy apartment buildings.

**Fig 2:**
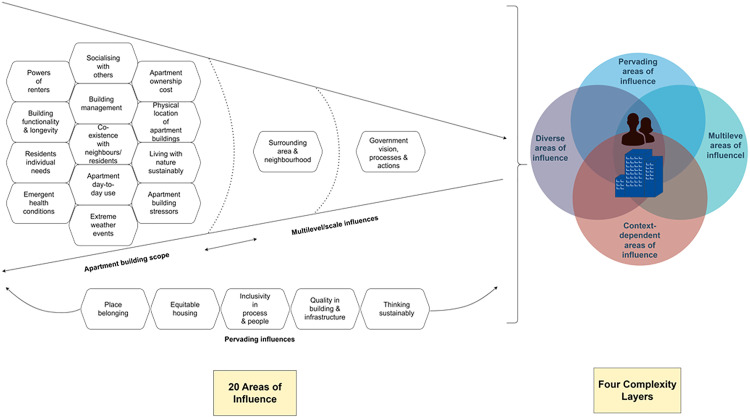
Conceptual model of health and wellbeing influences concerning high-density apartment living in Sydney.

We summarise this complex system in Fig 2 to highlight that the 20 themes represent influences that are: diverse, context-dependent, multilevel, and pervading. The following discussion section discusses these four complexity layers within the context of the 20 themes.

## Discussion and implications

This study documented residents’ perceptions of what influences their health and wellbeing concerning high-density apartment buildings within the case of Sydney, Australia. Overall, 20 areas of influence were identified from participants’ accounts. These influences emerged from residents, society, built environment, natural environment, stressors, and tenancy. A further synthesis of the 20 themes suggest that these influences on health and wellbeing are diverse, context-dependent, multilevel, and pervading.

### Diverse influences

Overall, participants identified 20 diverse influences affecting health and wellbeing in Sydney apartments, spanning residents, society, the built and natural environments, stressors, and tenancy. Unlike previous studies that addressed specific influences with limited scope [27–33], this research offers a broader, more defined and localised account of influences. This differs from the selected variable, subject, or resident group-focused model, where one or multiple areas of influence may or may not have been considered by each study. Further, this research presented surprising areas of influence that reflected the coupled human-environment perspective of health and wellbeing—an integrative approach to human health and the natural environment—encompassing ‘existing with nature sustainably’ and ‘thinking sustainably’. On the theme of ‘existing with nature sustainably’, the finding seems to demarcate and extend living with nature further, thus advancing prior research [30–33] by introducing this area of influence and demonstrating the diversity of health and wellbeing influences that we can uncover with a coupled human-environment systems approach.

Participants noted multiple dynamic factors within and beyond apartment buildings, reflecting the complexity of urban systems, where economic, environmental, political, social, and other systems, affect health and wellbeing in a messy, context-specific and complex way [6]. Such complex systems can ‘encircle’ health and wellbeing in habitats (such as apartment buildings), forming a causal web of influences [24]. In addition, an ecological perspective on health and wellbeing that considers interactions and interdependencies between residents (humans) as part of a living planet is absent in studies. The complex social and environmental context [24] interrelates all species, including humans, in the environment. Literally and not just metaphorically, such ecological notions situate humans as species among others that cohabit, evolve on and alter a dynamic planet [23]. When picturing this ecological view of health for apartment living, we can assume that the health of the public, including residents, becomes dependent on the natural world and co-existence with this world. This evolutionary thinking that informed the study design, and ultimately, the interview questions, makes conceptualising health concerning apartment living a continual outcome of many processes often expressed in dynamic systems as opposed to being static and a state of existence.

The diverse nature of influences suggests that targeting single or particular domains, sectors, or disciplines of research and practice separately is insufficient in addressing the complexities and future challenges concerning health and wellbeing and apartment living. Achieving healthy apartment living requires a holistic, cross-disciplinary, and cross-sector approach to future apartment developments, where elements from divergent fields and practice areas can be considered together. A further implication is that considering apartment building residents’ health and wellbeing as detached from nature can no longer be justified. Residents’ understandings call for a coupled resident-environment approach to influences of health and wellbeing. Such an approach is needed to promote health at the different stages of the apartment building development to deal with climate change and incorporate planetary health.

### Context-dependent influences

This study found unique, context-dependent influences on health and wellbeing perceived by Sydney residents, underscoring the role of context and local knowledge. Contextual aspects related to apartment buildings, residents, and the city of Sydney, as well as local and global conditions and events emerged as key influencers.

Conducting the research within Sydney apartments exposed locally specific areas of influence on health and wellbeing. Two themes ‘powers of renters’ and ‘apartment ownership cost’ stood out for their limited prior attention [27–33].

‘Powers of renters’ reflects power dynamics that can affect residents who live in apartment buildings in Sydney. Power dynamics, whether with real estate agents, landlords or other actors, may influence how much authority, control, and stability renters have over their situations and lives. The combination of such dynamics through tenure, whether social, legal, economic, or cultural, may expose households to physical and mental health impacts, including stress [7]. Hence, tenure security is associated with health through the material (physical) and the meaningful (social) aspects of housing. Compared to homeowners, renters’ rights and financial means may affect their ability to maintain and modify their homes, ultimately affecting their health and housing needs [7]. Concerning Sydney, comments over the lack of control by tenants, insecure occupancy, the powers of other actors (e.g., landlords and real estate agents) over renters, and the exclusion from building-related governance are recognised in the wider discourse [53,54]. For example, Easthope [55] emphasised the little attention given in Australia to the wellbeing of renters in policy and legislation due to renters’ lack of control over their dwellings.

‘Apartment ownership cost’ may have something to do with the costs incurred or required *to* own an apartment building in Sydney and the costs incurred *while* owning an apartment building in Sydney. The latter point appears to be linked to the cost of sharing with other owners through strata, the cost of repairs in apartments as well as the building, and future costs that occur after buying the apartment buildings without realising (e.g., the cost of solar electric wiring and cost of pre-signed agreements with service providers). While not reflected in the literature, the latest news and changes in the global and state economic outlook indicate an increase in the value of owning a house, including apartment buildings in Sydney. Recent project reports also discussed the defects in multi-unit strata titled developments as one of the significant financial burdens facing residents of apartment buildings in Sydney [56,57]. These reports pointed to the enormous physical and psychological stress and harm to owners and residents as a result of the defects. The extent of the costs incurred by owners because of apartment building defects is likely to be expensive. It may, depending on the apartment building history, lead to an increase in the annual strata budget shared by residents [57]. The finding aligns with these latest insights underscoring the role of apartment ownership cost in influencing health and wellbeing in Sydney.

Participants’ perceptions highlight that apartment residents and their coexistence with neighbours may influence health and wellbeing. The finding of ‘co-existence with neighbours/residents’ was absent in prior research [27,32,33]. Although, one study linked ‘good and friendly’ neighbours to residential satisfaction, their study focused mainly on design-related predictors [31]. This study identifies ‘co-existence with neighbours/residents’ as a distinct and stand-alone influencer of health and wellbeing that needs to be accounted for alongside other areas of influence, advancing our understanding in this area. Participants see interactions with floor neighbours and building residents as influential through socialisation and engagement. Coexistence can influence health and wellbeing via the building’s social environment, the engagement and levels of interaction between residents, and the care, privacy, respect and familiarity among apartment building residents. Broader literature also acknowledges neighbour relations, with a recent study by the Australian Housing and Urban Research Institute (AHURI) emphasising how living nearby and sharing spaces with neighbours can lead to annoyance or incidental interactions, both positive and negative [51]. By living nearby and sharing spaces with neighbours and residents, such interactions whether through nuisance or socialisation, can influence apartment building residents’ health and wellbeing.

This study also revealed the influence of global and local trends, particularly through the themes ‘emergent health conditions’ and ‘extreme weather events, ‘ tied to COVID-19 and the recent NSW weather events. Previous studies in Sydney, discussed the influence of COVID-19 lockdowns on families [33] or reduced apartment sizes [28], yet lacked consideration of ‘emergent health conditions’ as a standalone area of influence. By identifying this area of influence, this study expands the understanding of the type of health and wellbeing influences that need inclusion. Uncovering ‘emergent health conditions’ by participants is not a surprising finding given the timing of the study, which occurred during a global pandemic with enough time for participants to reflect on how acclimatising to COVID-19 has affected them while living in apartment buildings. However, what seems to be telling is the links participants made between ‘emergent health conditions’ and other influences or the way apartment buildings exposed residents to such conditions. Researchers discussed the momentum that the COVID-19 pandemic offered by focusing on ventilation, social distancing, and adaptable spaces [7]. Also, the health threats from infectious diseases that thrive when people are concentrated together require considering the urban and living environment instead of a narrow focus on healthcare [5]. Therefore, adequate infrastructure to address these health threats is critical for apartment living. In Sydney, concerns over the existing layout of apartment buildings and the connection with COVID-19 lockdowns was noted in a review of historical spatial layouts of apartment buildings in the city [58]. Adding to these concerns, this theme stresses the need to explicitly consider ‘emergent health conditions’ as influencers off health and wellbeing alongside other influences.

Another surprising finding within the context of Sydney, was the theme ‘extreme weather events’, attributed to global and local extreme weather events, including heatwaves and floods that swept Sydney in 2022 and beyond. As discussed earlier, there has been an accelerated change in the structure and function of our earth’s natural systems threatening human health locally and globally. Through environmental changes, such as climate change and urbanisation, residents may experience direct and indirect health effects, including heatwaves, floods, pollution exposure, and other threats. These threats, marked by surprise and uncertainty, are intensifying over time, prompting increasing public concern about climate change and environmental change [7,10]. This study reflects such perceptions and highlights a previously unidentified area of influence, emphasising the need to address pressing existing and future threats to health and wellbeing from climate change and global warming. Unlike prior Sydney-based studies that overlooked this area [27,31–33], this study recognises ‘extreme weather events’ as a distinct area of influence on health and wellbeing, expanding our understanding of factors associated with apartment living.

The themes related to local, residents, and global trends demonstrate the influence of context on health and wellbeing. Housing remains unique to every resident, household, and society [49]. Therefore, there is a need to consider the societal context in studies involving people and built environments, including housing, where the situation and the research project are embedded [49]. Using residents’ perceptions in different situations, the study has contextualised health and wellbeing influences relevant to residents and Sydney. Understanding local conditions is a critical first step in developing ideas for intervening in complex systems [7,49,59]. By identifying unique influences not previously reported, the study expands the literature on the type of factors residents associate with apartment living. A key implication from this finding is the necessity of consulting local knowledge and understanding context to create healthy apartment buildings in Sydney and beyond.

### Multilevel influences

Participant perceptions revealed themes extending beyond the apartment building level, including ‘surrounding area and neighbourhood’ and ‘government vision, processes and actions’. The latter theme on government vision, processes and actions is linked with the apartment building, surrounding area, and neighbourhood levels, and highlights the government’s role in shaping residential experiences. Surprisingly, previous Sydney-based studies did not consider this area of influence. While one study critiqued urban consolidation policies and their flow on health impacts on families in Sydney [33], another group of studies provided policy-specific health evidence to inform apartment design guidelines in three Australian states, including Sydney [27]. In contrast, this study identifies ‘government vision, processes and actions’ as a distinct area of influence, emphasising its consideration for health and wellbeing alongside other influences.

Apartment buildings are integral to the systems that influence health and wellbeing, shaped by how governments perceive, envision, plan, design, regulate, quality control, and integrate them. Ultimately, apartment buildings, including their surrounding areas and neighbourhoods, can be considered both a ‘product’ and a ‘byproduct’ of government vision, processes and actions. This multilevel view linking apartment buildings with their outside is rooted in systems and ecological thinking and makes it possible to understand the influence on health and wellbeing [8,19]. One of the features of complex systems that influence health in urban environments is the factors that operate at different levels of organisation (i.e., cities, neighbourhoods, homes, individuals) [60]. Therefore, health and wellbeing would manifest multilevel factors ranging from state and local government agencies to neighbourhoods, and apartment buildings. Such manifestation appears to transcend through the interrelated nature between the social processes of the government tiers (e.g., urban planning, policies and practices) and the physical environments (e.g., cities, neighbourhoods, and apartment buildings) underscoring the need to consider these influences collectively over time.

Uncovering ‘government vision, process and actions’ as an area of influence advances research by showing the role of multilevel influences as additional areas that we need to consider alongside other commonly studied influencers. The multilevel themes emphasise the interdependence of apartment buildings with its outside and show that we cannot create healthy apartment buildings in Sydney separately from the social processes taking place at government level or other scales (e.g., immediate surroundings, apartment building developments, neighbourhoods, and Sydney as a city). Apartment buildings cannot exist as isolated entities, separated from what happens within a resident’s surrounding area, neighbourhood, city, and government practices. Creating healthy apartment buildings require linking what occurs at a government level and the residents’ surrounding area together.

### Pervading influences

Participants’ perceptions reveal pervading areas of influence including ‘thinking sustainably’, ‘place belonging’, and ‘quality in building and infrastructure’. The theme ‘thinking sustainably’ reflects long-term, structural thinking about health and wellbeing through apartment development that supports both human and environmental health. Health and wellbeing emerge from the interconnections between human health and environmental health (both ecosystems and planetary), considered together at the level of apartment living. This perception of ‘thinking sustainably’ aligns with the broad view of sustainability concerning the built environment, emphasising the importance of supporting the needs of existing and future populations, whether spatially local or distant [8]. To achieve sustainable apartment living, urban health and environmental health agendas must be combined through the planning, design, and policy decision-making process, which study participants seem to convey through this finding. This differs from previous studies that did not consider or find this area of influence.

The finding ‘place belonging’ may be linked with the role of apartment buildings as places from a social sense, the complex connections between place, space, and health, or broader systemic societal issues. Participants provided insights into place as a social construct revealing its influence on health and wellbeing through sentiments like *“the feel of the place is just so light”*, *“very positive, very welcoming”*, *“it is a space that I feel like I want to be in”*. People develop an emotional attachment to or relationship with places, hence places can make them sick and affect their sense of wellbeing, including simply shaping how they feel about themselves [61]. Locationally, the remarks by one participant about the type of people and suburbs where they live seem to influence their health and wellbeing by making them feel *“quite welcome here”* compared to places in the city. For another participant, places closer to the city centre seem to lead to being *“lost and unnoticed”* compared to the suburb where they live. Such relationships between belonging and physical space show a complex, context-specific, and messy relation between place, space and health [61] making it difficult to understand residential environments. Some insights also indicate systemic societal issues related to community building, assimilation, and culture. Von Szombathely et al.’s separation of ‘households’ from ‘built structure’ factors in their model to signify the role of urban environments from a social (place) and physical (spatial) sense underscores the relevance of place belonging [62]. Whether a by-product of well-designed and planned places or an indicator of a hidden societal issue, ‘place belonging’ is a structural influencer of health and wellbeing that transcends and links with other areas of influence. Therefore, it must be regarded as a distinct influencer of health and wellbeing, unaddressed in prior studies.

Unlike other influences, which may or may not have been influenced by the interview questions, the theme ‘quality in building and infrastructure’ culminated from the data and transcended other areas of influence. Detecting quality is a feature of systems thinking. Therefore, registering the presence or absence of quality is imperative [63]. The variety of factors that participants found important appear to speak of an underlying issue about the construction standard of existing and new residential developments reflected through the standard of new builds and off-plan buildings in NSW, the quality inconsistency seen between different high-density developments in NSW as well as existing cases of bad building quality in NSW. These perceptions extended to the effectiveness and adequacy of infrastructure in Sydney, especially concerning the quality and adequacy of public transport and area/suburb parking. Capturing quality through this theme is understandable, given the issues reported in the media on poor quality apartment buildings in Sydney [64–66]. The Poor-quality of buildings prompted the NSW government to appoint a building commissioner in 2019 to address construction quality and restore trust in the industry. Researchers have also extensively documented the issue of defects and poor-quality construction in Sydney, NSW, and Australia [56,67–69]. One study associated defects with an increased risk of psychological distress and poorer mental health among apartment dwellers regardless of differences in tenure or area disadvantage in Sydney, Melbourne, and Perth [67]. Their study further showed a likelihood of renters and owner-occupier groups experiencing a range of stresses when seeking or negotiating remediation works. Such insights demonstrate the need to consider this area of influence in any decision-making on apartment living.

Uncovering the five pervading influences advances previous research by identifying structural areas of health and wellbeing influence previously overlooked. This study reveals and links together further types of health and wellbeing influences that were not distinguished, demarcated or considered as distinct areas that could influence health and wellbeing in earlier studies, not underlined by a coupled human-environment systems perspective of health and wellbeing. The findings suggest future research and practice on health and wellbeing, and apartment living, in Sydney or globally, must incorporate structural and ethical principles to achieve healthy environments. Further, influences like quality and place belonging, emphasise the importance of obtaining local knowledge as they may speak of underlying systemic issues.

Overall, this research addressed two aims, uncovering 20 influences and four complexity layers that offer crucial insights into residents’ perceptions through a coupled human-environment systems perspective. The first layer, ‘diverse areas of influence’ highlights the diverse nature of health and wellbeing influences tied to apartment living, shaped by the multiple dynamic entities and systems at work within/or outside the apartment building context and the intertwined human-nature connection. The remaining three complexity layers demonstrated the type of influences that residents of apartment buildings associated with health and wellbeing. Residents’ views revealed context-dependent, multilevel, and structural influences. These findings provide a holistic view of health and wellbeing influences, contextualised to Sydney, and illustrate complex views of health and wellbeing and apartment buildings as a system with components, interrelationships, and an open boundary.

### Limitations and implications for future research

This study reported the perceptions of 17 residents of high-density apartment buildings concerning influences on health and wellbeing in these built environments. Although this study prioritised depth and richness of data with the sample number of resident cases, a more significant sample and/or inclusion of other suburbs might have added more diversity to the data. Additionally, the study did not involve other stakeholders in understanding such a complex phenomenon of health and wellbeing and apartment living. This presents an opportunity for future work to be developed where residents’ understandings are put to other actors who play a part in developments that influence health and wellbeing. As a starting point, the findings suggest the need to include built environment and public health professionals and other actors, including property developers, strata management companies, real estate agents, policymakers, and private consultants. Finally, a small number of participants (2 out of 17) provided photographs following the interviews. Additional photographs with participants reflections may have added more depth and richness to the analysis, but we attribute the low numbers to the voluntary uptake of this additional data collection method.

Despite these limitations, this study reveals for the first time the complex ingredients that residents of apartment buildings perceive influencing their health and wellbeing with the use of a coupled human-environment systems perspective. This understanding is critical for research, practice, and policy given the rapid increase in high-density multi-unit developments, the numerous challenges facing urban health and the need to achieve sustainable urban development in Australia and abroad.

## Supporting information

S1 File Building selection criteria. (DOCX)

S2 File Apartment building participant selection criteria. (DOCX)

S3 Table Characteristics of all study participants. (DOCX)

S4 File Research design framework. (DOCX)

S5 File Preliminary interview guide and questions submitted to ethics committee. (DOCX)

S6 File Quality control and trustworthiness. (DOCX)

S7 Table Theme list and definitions. (DOCX)

S8 Table Additional participant quotes. (DOCX)
