# Supplementary material for: Exploring influences of health and wellbeing in Sydney’s apartment living: A qualitative study of residents’ perceptions
Source: PLoS One. 2025 Aug 6;20(8):e0329879. doi: 10.1371/journal.pone.0329879 (PMC12327653; doi:10.1371/journal.pone.0329879)
Supplement: S1 File — (DOCX) [file pone.0329879.s001.docx]

Extensive site visits were conducted by (TA) across Sydney before recruiting participants to establish suitable apartment buildings that fit the following four criteria:

1. Apartment buildings located in three diverse geographic areas of Sydney with different climates: near the coast of Sydney, towards the middle near the Parramatta River, and further inland towards the far west of Sydney.

2. Apartment buildings within a fifteen-minute walk radius to the nearest transit nodes/transport hubs and business centres.

3. Apartment buildings that are four storeys and above with the inclusion of residents from buildings of four to eight storey heights, nine to19 storey heights, and 20 or more storey heights to reflect the predominant building heights in the Sydney Market.

4. Apartment buildings built since 2006 to coincide with the start of the metropolitan strategy planning adopted by all Australian states, including NSW during the 2000s.

The selection of the three geographical locations in Sydney was based on existing spread of apartment stock across Sydney’s metropolitan area in the form of higher density housing around strategic centres with mixed-use transport orientation [1, 2]. The planned and projected increase in housing stock to cover the identified 725,000 additional homes by 2036 is anticipated across different geographical locations around Sydney (Greater Sydney Commission, 2018). In addition, buildings are distributed across Sydney, characterised by climate variations ranging from less hot rainy with more tree coverage climate patterns closer towards the coast to hot rainy with less tree coverage towards the west [3]. As a result, it was deemed appropriate to select buildings from across Sydney with equal sampling numbers of residents recruited from the coast, towards the middle near the Parramatta River, and the far west.

A selection of suburbs with existing buildings were visited to verify the four criteria. Suburbs were selected based on observations, word of mouth, supervisor recommendations or last-minute additions while visiting suburbs. The first author (TA) then selected suitable buildings based on site visit notes and internet checks to confirm access to building letterboxes, building age, building tenure type, building storey level, and any relevant history.

**References**

1. Easthope H, Crommelin L, Troy L, Davison G, Nethercote M, Foster S, et al. Improving outcomes for apartment residents and neighbourhoods. AHURI Final Report, 329. 2020. doi: 10.18408/ahuri-7120701.

2. Randolph B, Tice A. Who lives in higher density housing? a study of spatially discontinuous housing sub-markets in Sydney and Melbourne. Urban Stud. 2013;50(13):2661-81. doi: 10.1177/0042098013477701.

3. Greater Sydney Commission. A Metropolis of Three Cities – The Greater Sydney Region Plan. Greater Sydney Commission. 2018. <https://www.planning.nsw.gov.au/Plans-for-your-area/A-Metropolisof-Three-Cities/A-Metropolis-of-Three-Cities>
