# Supplementary material for: Exploring influences of health and wellbeing in Sydney’s apartment living: A qualitative study of residents’ perceptions
Source: PLoS One. 2025 Aug 6;20(8):e0329879. doi: 10.1371/journal.pone.0329879 (PMC12327653; doi:10.1371/journal.pone.0329879)
Supplement: S2 File — (DOCX) [file pone.0329879.s002.docx]

The following minimum criteria were used to include participants. Participants were selected based on whether all the following inclusion criteria were fulfilled:

1. At or over the age of 18.

2. One participant representing each household.

3. An independent owner or renter (i.e. if participating will not pose a conflict of interest based on their backgrounds).

4. Able to read and understand English.

5. The selection of at least one resident from each of Sydney’s most dominant high-density building submarket groups.

Participant sampling involved selecting at least one resident from Sydney’s most dominant apartment submarket groups. According to previous research, Sydney apartment residents are heterogeneous with distinctive submarkets [1, 2]. Accordingly, we aimed to recruit at least one resident from each submarket group in Table S2.1 below anywhere within the three geographic locations in Greater Sydney.

**Table S2.1:** Participant selection criteria based on the most dominant apartment building submarket groups in Sydney, adopted from [1,2]

| Most dominant high-density submarket groups in Sydney | Description |
| --- | --- |
| Group 1 | A single resident or couples belonging to the age group (25-34 years) with a weekly income above A$1499 |
| Group 2 | A resident belonging to the age group (18-34 years) either living alone or sharing with others with a weekly income below A$1499 |
| Group 3 | A student in lone or shared group households belonging to the age group (18-24 years) |
| Group 4 | A family with children under the age of 18, born overseas with a weekly income below A$1499 |
| Group 5 | Lone households of residents aged 55 years and above with no children |
| Group 6 | A resident belonging to the age group (35-54 years) with a weekly income of over A$2500 |
| Group 7 | Other |

**References**

1. Easthope H, Crommelin L, Troy L, Davison G, Nethercote M, Foster S, et al. Improving outcomes for apartment residents and neighbourhoods. AHURI Final Report, 329. 2020. doi: 10.18408/ahuri-7120701.

2. Randolph B, Tice A. Who lives in higher density housing? a study of spatially discontinuous housing sub-markets in Sydney and Melbourne. Urban Stud. 2013;50(13):2661-81. doi: 10.1177/0042098013477701.
