# Supplementary material for: Exploring influences of health and wellbeing in Sydney’s apartment living: A qualitative study of residents’ perceptions
Source: PLoS One. 2025 Aug 6;20(8):e0329879. doi: 10.1371/journal.pone.0329879 (PMC12327653; doi:10.1371/journal.pone.0329879)
Supplement: S3 Table — (DOCX) [file pone.0329879.s003.docx]

| Interview no | Participant code (Fictitious name) | Greater Sydney geographic area | Suburb | Total building storey level | Apartment level | Tenure | Household | Age | Gender | Study criteria group |
| --- | --- | --- | --- | --- | --- | --- | --- | --- | --- | --- |
| 1 | Lou | Coastal | Redfern | 18 | 10 | Renter | Couple (professional working couple) | 25-34 age group | Female | Group 1 |
| 2 | Steffan | River | Rhodes | 19 | 5 | Renter | Single | 35-54 age group | Male | Group 6 (weekly income above $2500) |
| 3 | Rence | Coastal | Marrickville | 7 | 5 | Owner | Couple (professional working) (no children) | 35-54 age group | Male | Group 6 (weekly income above $2500) |
| 4 | Lux | Inland | Penrith | 10 | 8 | Owner | Single/retiree | 64 | Female | Group 5 |
| 5 | Rani | Inland | Penrith | 6 | 2 | Renter | Couple (no children) | 35-54 age group | Female | Group 7 (other) |
| 6 | Valerie | Inland | Penrith | 10 | 6 | Renter | Couple (no children) | 25-34 age group | Female | Group 1 |
| 7 | Trevor | Inland | Penrith | 9 | 8 | Renter | Single | 50 | Male | Group 7 (income around $1100 a week) |
| 8 | Ada | River | Parramatta | 16 | 10 | Owner | Couple (no children) | 41 | Female | Group 7 (both younger than 54 with combined income of over $2500/week) |
| 9 | Sandra | Coastal | Arncliffe | 5 | 2 | Renter | Sharing with a friend/roommate | 15-34 age group | Female | Group 2 (with a weekly income below A$1499) |
| 10 | Ani | Coastal | Erskineville | 8 | 7 | Owner | Couple with one child under 18 (18 months old) | 34 | Female | Group 7 (born overseas, weekly income above $1400) |
| 11 | Jon | Coastal | Erskineville | 6 | 6 | Owner | Couple/retirees | 70 | Male | Group 5 |
| 12 | George | Coastal | Erskineville | 8 | 6 | Owner | Couple (a male couple) | 58 | Male | Group 7 (gay male couple of 50 and 58 with no kids) |
| 13 | Yonas | Coastal | Zetland | 9 | 8 | Renter | An international research student (sharing with a flatmate) | 31 | Male | Group 2 (~$1200/2 weeks) |
| 14 | Matt | Coastal | Erskineville | 8 | 4 | Owner | A single resident working full-time | 30-40 | Male | Group 1 or 6 (depending on age) |
| 15 | Jane | River | Parramatta | 46 | 13 | Owner | Single/retiree | 68 | Female | Group 5 (retired) |
| 16 | Martin | Coastal | Erskineville | 8 | 4 | Renter | Single parent with a 13 year old child | - | Male | Group 7 (Single parent with 13 year old child) |
| 17 | Ryan | Coastal | Crows Nest | 5 | Not provided | Renter | Single resident working full-time | In mid 30s | Male | Group 6 |
