# Supplementary material for: Exploring influences of health and wellbeing in Sydney’s apartment living: A qualitative study of residents’ perceptions
Source: PLoS One. 2025 Aug 6;20(8):e0329879. doi: 10.1371/journal.pone.0329879 (PMC12327653; doi:10.1371/journal.pone.0329879)
Supplement: S4 File — (DOCX) [file pone.0329879.s004.docx]

The novel framework in Fig 1 lays out preliminary conceptual assumptions that guided the study’s research questions, participant interview questions, and data analysis and synthesis. It draws on multiple concepts informed by theoretical approaches and other findings from the literature.

Approaches underlined with complex systems and ecological thinking of health and wellbeing have seen a recent surge in the literature in order to understand and address the systemic factors and problems influencing health in urban and living environments. Although, not apartment building specific, such approaches including those by Diez Roux [1], Pineo [2], and von Szombathely et al. [3] have been proposed as useful in understanding health and wellbeing influences concerning urban environments.

These recent development in healthy urbanism approaches reflect the concepts of systems thinking, ecological public health, healthy urban development principles, and the main areas (domains) of health and wellbeing influences behind the framework. The main conceptual and relational boundaries and concepts behind the framework are summarised below:

- Health and wellbeing

Health and wellbeing are considered the main subject of the framework representing the health and wellbeing of an urban population, as envisaged by von Szombathely et al. [3], examined within the apartment building context. Health and wellbeing interlink human health (both the physical and the mental) and the health of ecosystems and the planet, as conceptualised by Pineo (2). They represent the physical and mental health outcomes of diverse factors (e.g., individual, social, built, and environmental) and their relationships [3], as well as the continual and adaptive outcomes of many processes (e.g., structural barriers to health, the human health impacts of environmental degradation, and the health impacts of new developments and regenerated areas) where health status becomes a function of space and time variations when studied in specific contexts [2].

- System and Study Boundary (Apartment Buildings)

The contextual focus—the orange-coloured oval shape shown in Fig 1 is defined within the apartment building, setting a clear boundary between the concepts under examination and the limits of the study. However, interactions are not strictly confined within a boundary represented by apartment buildings. Systems and ecological thinking, approach, and analysis posit that interactions co-occur transcending spatial scales and levels of societal organisation where ultimately, the passage of time and the actions of institutions, social movements and social actors frame these interactions between residents and apartment buildings [2-9]. The framework’s cone shape acknowledges these multiple scales and levels of influence on health and wellbeing. Within the context of this research, institutions, social actors, and movements can be viewed as providing key overarching values and norms, such as equity and sustainable development, and can be viewed as organisational institutions that influence policies affecting the urban environment and health.

- A Coupled Human-Environment Systems View of Factors

The framework has a coupled social-ecological systems view of factor interactions affecting health and wellbeing within urban environments, reflecting von Szombathely et al.’s binary view of humans and the environment, where factors relevant to apartment living can be addressed within this complex system. Within the binary structure, von Szombathely et al.’s four domains of individual, society, morphology, and environmental stressors intersect different factors where dynamic non-linear interrelationships function at multiple levels (at the cellular, molecular, individual, population and societal levels of organisation) [3,7]. These factors and domains constituted the main components that informed and directed the research questions rather than illustrating rigid interrelationships between the factors and domains themselves. The factors reflect bidirectional interrelationships between individual characteristics, lifestyles, and behaviour, place-based environmental contexts, and geographical variations in health outcomes [10]. The framework also builds on von Szombathely et al.'s social and environmental stratification concept [3]. For example, any effects from the ‘built environment’ are separated from ‘households’ to consider the building quality and usage. Also acknowledged in the framework are design and planning factors relevant to the health of ecosystems and the planet without separating them from those relevant to the apartment building context.

- Overarching principles

The conceptual framework is further underpinned by the overarching principles of equity, inclusion, and sustainability inspired by Pineo and von Szombathely et al. frameworks [2-3]. Equity relates to access to health-promoting urban environments and reducing barriers to access and differences in risk exposures that result in inequalities in health and risk distribution in groups. Inclusion relates to satisfying human-related health conditions and differences (e.g. race, gender, age, disability) and the participation of communities and local citizens in upcoming developments and their design and shape. Sustainability is balancing economic, social, and environmental goals and achieving equity and environmental justice for the planet and future generations. These core principles underpinned the framework in terms of the conceptual exploration with participants and provided the ethical framing of the study design.

**References**

1. Diez Roux AV. Conceptual models and frameworks for understanding the links between urban environments and health. In: Lovasi GS, Diez Roux AV, Kolker J, editors. Urban public health: a research toolkit for practice and impact: Oxford University Press; 2020. p. 56-76.

2. Pineo H. Towards healthy urbanism: inclusive, equitable and sustainable (THRIVES)–an urban design and planning framework from theory to praxis. Cities Health. 2020;6(5):974-92. doi: 10.1080/23748834.2020.1769527.

3. von Szombathely M, Albrecht M, Antanaskovic D, Augustin J, Augustin M, Bechtel B, et al. A conceptual modeling approach to health-related urban well-being. Urban Sci. 2017;1(17):1-18. doi: 10.3390/urbansci1020017.

4. Berkes F, Folke C. Linking social and ecological systems for resilience and sustainability. In: Berkes F, Folke C, Colding J, editors. Linking social and ecological systems: management practices and social mechanisms for building resilience. Cambridge, U.K.: Cambridge University Press; 1998. p. 1-27.

5. de Leeuw E, Green G. The logic of method for evaluating healthy cities. In: de Leeuw E, Simos J, editors. Healthy cities the theory, policy, and practice of value-based urban planning. New York, NY: Springer New York; 2017. p. 463-489.

6. Rayner G, Lang T. Ecological public health: reshaping the conditions for good health. Abingdon, Oxon: Earthscan; 2012.

7. Sarkar C, Webster C. Urban environments and human health: current trends and future directions. Curr Opin Environ Sustain. 2017;25:33-44. doi: 10.1016/j.cosust.2017.06.001.

8. World Health Organization. A Conceptual Framework for Action on The Social Determinants of Health [internet]. World Health Organization; 2010 [cited 2024 Aug 6]. Available from: https://www.who.int/publications/i/item/9789241500852.

9. Krieger N. Proximal, distal, and the politics of causation: what’s level got to do with it? Am J Public Health. 2008;98(2):221-30. doi: 10.2105/AJPH.2007.111278.

10. von Szombathely M, Bechtel B, Lemke B, Oßenbrügge J, Pohl T, Pott M. Empirical evidences for urban influences on public health in hamburg. Appl Sci (Basel). 2019;9(11):2303. doi: 10.3390/app9112303.
