# Supplementary material for: Exploring influences of health and wellbeing in Sydney’s apartment living: A qualitative study of residents’ perceptions
Source: PLoS One. 2025 Aug 6;20(8):e0329879. doi: 10.1371/journal.pone.0329879 (PMC12327653; doi:10.1371/journal.pone.0329879)
Supplement: S5 File — (DOCX) [file pone.0329879.s005.docx]

The design of the interview guide was formulated based on Arthur and Nazroo (2003, Chapter 5), Patton, (2015, Chapter 7), and Tolley et al. (2016, Chapter 4) [1-3]. The interviews with residents of apartment buildings in Sydney will follow a semi-structured, open-ended interview style. Based on the goals and aims of this study, this qualitative study will combine and revolve around different question types: opinion/value question, knowledge generation question, and behaviour question.

The interview guide includes general questions and topics that can be covered. Qualitative research questions could evolve, change and grow based on the iterative nature of qualitative research. This is also anticipated in a naturalistic inquiry design as the interviewer understands the situation more entirely and discovers new pathways for questioning (Patton, 2015, Chapter 7). However, consistency and vigilance will be maintained to ensure a clear link between the questions and the research purpose.

Participants will not be asked direct questions about their health and wellbeing while living in apartment buildings. Instead, all questions and areas of inquiry will focus on understanding the participants’ point of view of what would make apartment buildings enhancive of health and wellbeing.

This draft interview guide is designed to be flexible and to explore topics earlier or later during the interview.

The following represents an outline of areas that the research student could cover:

**INTRODUCTION**

- Obtain Verbal consent by reading the verbal consent form to participants.
- Proceed to record the interview using Microsoft Teams or an audio recorder.
- Explain to the participant that:
- This study aims to improve residents’ health and wellbeing and achieve equitable, inclusive and sustainable buildings.
- A building is a permanent structure that has a roof that houses your apartment.
- This study examines residential buildings only, which are four storeys and above.
- When we refer to the building, we refer to anything you can feel, touch, smell, hear, see, and think of from the entrance to the point of exit, including the immediate surroundings of the building.
- An apartment is a structure where you reside with rooms, a cooking facility, and a bathing and shower facility within your residential building. Your apartment includes everything you can smell, touch, feel, hear, see, and think of that extends from your apartment door to the balcony and even views from the balcony.
- And some of the questions are purposefully vague so you (the participant) can respond in any way that makes sense to you.

1 PRESENT CIRCUMSTANCES

- Review the inclusion criteria checked by residents on the participant information sheet (ETH21-6605 Participant information sheet consent-Residents).

2 KEY QUESTION – understandings/meanings

- In your opinion, what does being healthy and well in an apartment building mean to you?

Probes

- Tell me more, if you will, about your thinking on that? OR
- Can you elaborate on your answer?

3 KEY QUESTION – knowledge generation

- So reflecting deeper, in your view, what makes a healthy apartment building?

Areas

- Individual aspects?
- Social aspects?
- Building or apartment structure aspects?
- The feel/sense of place of apartment buildings?
- Environmental stressors?

SUB-QUESTION (if residents do not elaborate on this area)

This study aims to improve the health and wellbeing of residents of apartment buildings and achieve equitable and inclusive buildings. *Equitable buildings mean residents can access buildings that promote health and not have to deal with barriers to this or experience any differences in risk exposure between residents. Inclusive buildings are ones that accommodate for differences in age, race, gender, disability or other individual factors Inclusion also involves residents, local citizens and communities being able to participate in new developments through their design, shape and decision-making process.*

- So reflecting back on this, what is your opinion about equitable and inclusive buildings?

SUB-QUESTION (if residents do not elaborate on this area)

This study considers human health as linked with and part of the health of the natural environment and our planet. The natural environment refers to both living things (such as plants, animals, microorganisms, trees, and humans) and non-living things (atmosphere, hydrology, geology, natural resources).

- So reflecting back on this, what is your opinion about the relationship between the natural environment, health and wellbeing and your apartment building?
- How do you see this relationship enhancing health and wellbeing within apartment buildings?
- This study also aims to achieve sustainable buildings. Reflecting back on this, what is your opinion about sustainable apartment buildings?

FOLLOW-UP QUESTIONS – Behaviour/attitudes

Depends on the context and only if participants emphasise a specific decision/action/behaviour during the interview (e.g. I will not live in apartment buildings again).

- A few minutes ago you mentioned….What influenced your decision? OR What influenced your beliefs/attitude?

**END**

- That covers the things I wanted to ask about today. Anything you would like to add OR what should I have asked you about that I didn’t think to ask?
- And what should I pay most attention to out of all the things we talked about today—or maybe some topics we missed? What should I think about when I read your interview?
- Reiterate confidentiality agreements, how the data will be used, and sending copies of reports/results.
- Residents will be informed about the next steps:
- They will receive a further email should they wish to complement the interview with narrated photographs. This email will be sent following the interview with the interview transcript for their information and review. A grocery gift voucher will be delivered via the same email as an e-voucher in the first instance. The gift voucher can be posted if residents express their desire for this option; and
- They may be re-contacted in the future for further information if needed, such as in the case of missing information, clarifying certain aspects raised during the interview, or for any administrative or logistical reasons deemed important to the participant.
- Ask if residents have any questions.
- Bring the interview to a close.

**References**

1. Arthur S & Nazroo J. Designing Fieldwork Strategies and Materials. In Ritchie J & Lewis J, editors. Qualitative research practice: a guide for social science students and researchers. Sage Publications; 2003. p. 109-138.
2. Patton MQ. Qualitative research & evaluation methods: integrating theory and practice. 4th ed. Sage Publications, Inc; 2015.
3. Tolley, EE, Ulin PR, Mack N, Robinson ET, Succop SM. Qualitative methods in public health: a field guide for applied research. 2nd ed. John Wiley & Sons, Incorporated; 2016.
