# Supplementary material for: Exploring influences of health and wellbeing in Sydney’s apartment living: A qualitative study of residents’ perceptions
Source: PLoS One. 2025 Aug 6;20(8):e0329879. doi: 10.1371/journal.pone.0329879 (PMC12327653; doi:10.1371/journal.pone.0329879)
Supplement: S6 File — (DOCX) [file pone.0329879.s006.docx]

The study followed the four criteria of credibility, dependability, confirmability, and transferability by Guba and Lincoln [1,2]. Each of these are discussed below with the strategies used to ensure research rigor and transparency:

- **Credibility -** the extensive field visits, residents’ interviews and photographs analysed using template analysis involved repeated and substantial engagement with different cases of residents of apartment buildings. The methodology process is detailed in the methods section of this manuscript with thick descriptions of diverse participant themes with discrepant findings presented in the results section. In qualitative research, credibility is also linked with the use of multiple data sources. This was addressed through the field visits and semi-structured interviews with divergent residents of different apartment buildings in Sydney and the inclusion of photographs as another reflection method. Participants were allowed to review the interview transcripts and make changes if they wanted to amend what they said or clarify connotations. On regular basis, the first author TA consulted and debriefed JP and EM on the process with some changes implemented accordingly. Any possible biases were kept ‘in-check’ through self-reflections and documentation.
- **Dependability** – the rationale behind the choice of methods was philosophically driven by complex views of the study subject. The use of an audit trail of the analysis process ensured a logical and documented research process.
- **Transferability** – the methods and results sections of the manuscript outlines the purposeful sampling strategy used and the thick descriptions of the final themes which demonstrate contextual detail to the reader and future researchers in this area.
- **Confirmability** – the reasons behind the discussed methodological, theoretical and analytical choices are provided in the manuscript.

Further, to ensure the trustworthiness of the qualitative study, triangulation is also discussed as an additional strategy implemented to cross-check information and conclusions systematically [1].

- **Triangulation** – as many data sources as possible and practicable were used to examine data at varying times at different apartment buildings in Sydney with different apartment levels, buildings heights, area profiles, suburbs, and building locations. In alignment with the systems thinking lens, a heterogeneous mix of residents of apartment buildings from diverse backgrounds was used to capture multiple perspectives and experiences. In addition, multiple data were used to look at the data methodologically. While theoretically, the literature review behind the study explored a range of cross-disciplinary theories and concepts which framed the study in context and broadened the relevance of the research study.

**References**

1. Bloomberg LD, Volpe M. *Completing your qualitative dissertation: A road map from beginning to end*. 4th ed. Thousand Oaks, CA: SAGE Publications; 2019.
2. Nowell LS, Norris JM, White DE, Moules NJ. Thematic analysis: Striving to meet the trustworthiness criteria. *Int J Qual Methods*. 2017;16(1):1-13. doi:10.1177/1609406917733847.
