# Supplementary material for: Exploring influences of health and wellbeing in Sydney’s apartment living: A qualitative study of residents’ perceptions
Source: PLoS One. 2025 Aug 6;20(8):e0329879. doi: 10.1371/journal.pone.0329879 (PMC12327653; doi:10.1371/journal.pone.0329879)
Supplement: S7 Table — (DOCX) [file pone.0329879.s007.docx]

| 20 Influences of health and wellbeing (themes) | Definitions |
| --- | --- |
| Apartment building stressors | *Captures factors that speak of strain and tension that can affect residents of apartment buildings through several areas. Sub-themes emerging from this pattern indicate stressors related to noise, carparking, building residents, views from one's apartment, the building itself and its surroundings, construction issues, the apartment itself, and those concerning residents themselves.* |
| Apartment day-to-day use | *Refers to the various aspects of apartment living that speak to its functionality and dimensions of daily use by residents of apartment buildings. Specifically, these patterns are reflected through several aspects, including: apartment windows, exposure to sunlight, balconies, apartment space and size, apartment design, apartment access, apartment appliances, views, insulation and soundproofing, apartment ventilation, and apartment layout.* |
| Apartment ownership cost | *Reflects a clear pattern around factors related to the expense of owning apartment buildings incurred by residents.* |
| Building functionality and longevity | *Refers to wider aspects relating to apartment building design, layout, use and longevity. This area of influence seems to be captured based on prominent sub-patterns around (building design, carparking, building lifts, building access, fire safety, and building longevity), that speak to the quality of buildings and how useful, practical, safe and purposeful they are for residents daily as well as their ability to last long in the future.* |
| Building management | *Refers to wider aspects that relate to the running and management of an apartment building. This area of influence is captured based on six prominent sub-themes related to the handling of building rules, living under strata, management personnel, communication with residents, building management style, and areas of management.* |
| Co-existence with neighbours/residents | *Captures a distinct pattern around the dynamics of how a resident exists, be, socialises, and lives alongside other neighbours and residents of apartment buildings. The pattern speaks to social aspects related to socialisation, engagement, levels of interactions with residents, care, privacy, respect, knowing others and the building's social environment level.* |
| Equitable housing | *Captures various aspects related to the importance of fairness and justice for residents living in apartment buildings and beyond.* |
| Emergent health conditions | *Captures a pattern around the prominence of diseases, including COVID-19 and how residents connect these factors with potential exposure in an apartment building.* |
| Extreme weather events | *Captures a pattern around weather events considered extreme or unusual that have already or could potentially affect residents unexpectedly.* |
| Government vision, processes and actions | *Captures how residents perceive the role of government in influencing how residents experience residential developments. Through their foresight, processes and actions, different tiers of government (whether local, state or federal governments) ultimately influence health and wellbeing through apartment buildings. Various patterns reflect the role of government in influencing health through apartment buildings: planning vision, integrative planning, actions through regulations, acting on the environment, quality control, and overarching city vision.* |
| Inclusivity in process and people | *Shows a pattern around the inclusion of different residents and their ideas through both the process of creating apartment buildings—where residents get to be heard and represented—as well as in accommodating resident differences whether in culture, sexual orientation, disability, age or life stage.* |
| Living with nature sustainably | *Refers to wider factors that speak to the need for living and existing with nature in sustainable ways through sustainable design and planning of buildings, as our health is ultimately linked to and dependent on the health of the natural environment and our planet.* |
| Place belonging | *Captures a variety of aspects that shows the importance of a resident feeling at home or belonging to where they live while living in an apartment building. The theme talks to the role of apartment buildings as a place in a social sense rather than a physical one.* |
| Powers of renters | *Captures a distinct pattern around factors that affect how much control and authority renters have when renting in their apartment building.* |
| Physical location of apartment buildings | *Explores a prominent pattern that revolves around the physical and geographic location of apartment buildings expressed in various ways throughout the dataset.* |
| Quality in building and infrastructure | *Explores a wide range of factors that speak to the importance of well-built apartment buildings and satisfactory and effective infrastructure alongside residential developments.* |
| Residents’ individual needs | *Captures a core idea related to the various factors the resident considers preferential or necessary for a satisfactory life. This category highlights the importance of considering resident differences along with their needs and desires when it comes to how they interact and live in apartment buildings. It encompasses patterns that revolve around: individual wishes and wants, personal preferences, sociodemographics, attitudes, female safety, lifestyle, personal needs, and medical constitution.* |
| Thinking sustainably | *Demonstrates the necessity and significance that residents place on thinking about apartment buildings in sustainable ways. Ways that ensure any apartment buildings benefit people and the environment and achieve ecological balance.* |
| Socialising with others | *Captures a pattern related to residents' socialisation and how they connect with members of society whether one's own family, relatives, friends or those outside one's social circle.* |
| Surrounding area and neighbourhood | *Talks to the importance of considering factors that could fall outside the immediate apartment building remit and within the building's surrounding area of development or neighbourhood. A diverse set of factors fall under many sub-patterns reflecting this area of influence including: neighbourhood stressors, the design of residential developments, the neighbourhood influence, existing with the natural environment, and area walkability.* |
