# Supplementary material for: Exploring influences of health and wellbeing in Sydney’s apartment living: A qualitative study of residents’ perceptions
Source: PLoS One. 2025 Aug 6;20(8):e0329879. doi: 10.1371/journal.pone.0329879 (PMC12327653; doi:10.1371/journal.pone.0329879)
Supplement: S8 Table — (DOCX) [file pone.0329879.s008.docx]

| **Theme** | **Additional examples** |
| --- | --- |
| Living with nature sustainably | On living and existing with nature, one participant (Sandra) and other participants showed the presence of indoor plants in the apartment as an influencing factor on their health and wellbeing:  *“[…] we put a lot of like plants in our apartment to kind of bring that nature element to it because I think that just improves my mental health so much.”* (Sandra)  Participant (Sandra) agreed with the notion that human health is linked and is dependent on the health of the natural environment and the planet. In her additional remarks she reiterated the influence of having indoor plants on their health and wellbeing:  *“OK, so I think it's a very big relationship. I can definitely agree that it's. Umm. Kind of combined together, it's not separate I guess humans and wellbeing plus the environment, so I think it's a big impact on how we live. Umm. I guess like I said before, having plants here, I've noticed for us as being a big impact and uh, I guess just making it feel different in the room, like it makes you feel a little bit happier, having life around you […].”* (Sandra)  By being on the strata committee, participant (Jon) pushed for the provision of electric car charging points and alluded to the role of proactivity on sustainability in the building by undertaking the NABERS Australian environmental rating scheme despite the challenging processes involved:  *“I don't know if you've heard of it, but our environmental rating, NABERS.[…], it rates, sort of greenness and the strata have done quite well on that and so much so that we were entered into some sort of process that allowed us to get a free consultation on other things we could do so, so that's been good and we've both been supportive of that and but then again but compared to having our own house where we could just drive electric car into the garage and plug it in. Yes (laughter). It's a bit more of a process…”* (Jon) |
| Powers of renters | Please refer to what is in the results section |
| Apartment ownership cost | Please refer to what is in the results section |
| Co-existence with neighbours/residents | Some participants also talked about socialisation with their neighbours. For instance, one participant described how they would happily socialise with their neighbours whom they socialised with before:  *“…we're lucky we get on really well with our neighbours but we actually didn't speak to them for like the first year of them being here because we were just those awkward neighbours that never ran into each other or just said hello in the hallway. And it was only until, because they'd had a baby, and then it was only when we had a baby that we we talked and like now we went to (ANON) 2nd birthday and we went, you know, for the gender reveal party, you know, for their new kid and like we yeah, like. And I was messaging her today and I'm gonna look after her cats and actually it's been really nice and they are people that we would like happily be friends with and socialise with and it was very fortuitous but it happened it's so rare and yet like we're all just in our like little lonely (laughter) like block so you could imagine that need for connection and if loneliness is becoming, you know of a trend which is a shame, the importance of community that sits around that.”* (Ani)  In the excerpt above, the participant talked about the importance of socialising with neighbours and also linked this factor with loneliness in apartment living. |
| Emergent health conditions | Please refer to what is in the results section |
| Extreme weather events | Please refer to what is in the results section |
| Government vision, processes and actions | Please refer to what is in the results section |
| Thinking sustainably | Participant (Steffan) further gave credence to sustainable thinking by commenting on the co-benefits offered to the local area where he lives and the natural environment:  *“…it goes to show that sometimes development can be good for the local area, the environment. And they've actually got, like, a water bird refuge around the corner. Now that would have been all polluted as well.”* (Steffan)  By rehabilitating the land in an area that was polluted with toxic waste, the suburb not only has a rehabilitated land but now is home to a water bird refuge and becomes a home for many residents, including participant (Steffan). |
| Place belonging | In addition to location, participant (Valerie) further associated sense of belonging with other factors related to the apartment building, such as views from her apartment window and presence of natural sunlight:  *“Yeah, like the place that I was at before this, very nice building in a very nice sort of more of a wealthy area, I guess very close to the city. I felt very negative being in there. I always felt like there was something on my shoulders weighing me down. And I'm not going to know for sure, but I really did think and attribute that to like the lack of light and not being able to see outside as much as we can here. But being here, the feel of the place is just so light. And yeah, very positive, very welcoming. It's a space that I feel like I want to be in. I'm never thinking, oh I have to go home, or oh I have to do that. There's no negative connotations around it. Like I said, I've lived in places where you did, not dread coming home, but it was just this feeling and it wasn't so positive, or you felt a bit boxed in, or you felt a bit claustrophobic or dark, and I just didn't feel like there were places where I could personally grow or feel safe and like completely hole in and feel okay there.”* (Valerie)  Participant (Valerie)’s further insights also showed links between sense of belonging with the type of people in her neighbourhood compared to those who live towards the city of Sydney area:  *“…the types of people here, I think, towards the city, you get a lot of very busy people. Potentially, stereotypically maybe a little bit more selfish types. Like, I could go for a walk here and every second person will smile and say good morning. If I went for a walk around an oval in Redfern, chances are no one's gonna do that, not to the same capacity. So, you really do feel quite welcomed here. You don't feel invisible, which you do, and I have found feeling in places toward the city; it is very much a place where you can get lost and unnoticed. Whereas yeah out here - I don't know if that's, um, yeah, maybe the location, different types of people that are around, it's a little bit more slower pace, which is nice.”* (Valerie)  Further excerpt by participant (Ani) alluding to how difficult it is to make friends in Australia and the degree of difficulty to belong, which also seems to influence her sense of belonging:  *“…so I feel like there's definitely kind of gaps there and then particularly from like mum slash primary caregiver of…a toddler like our mother's groups didn't really, like we met up a couple of times, but it felt like everyone kind of already had their group and so…I was then quite good friends with one British lady, another British lady and one other mum…happens to live across the road in the (ANON) building. But then the British one who I got on really well with and, and felt the same. That like people didn't necessarily or often Australians, weren't making too much of an effort to go out their way to, like, make new, umm make new friends and you know they've got friends, they got friends with babies, they don't necessarily need any more friends with babies. Yeah, like she felt it as well but then she ended up moving back to England...to again be closer to family... So yeah, you definitely like, I feel like it's becoming more and more of an effort to be able to find a sense of belonging, like even coming to Australia, to be honest like I think it took me a good two to three years to feel like I had friends of my own and like people I can…you know, see umm, I think I'm still…I think what we don't have is like that big group of friends.”* (Ani) |
| Quality in building and infrastructure | Participant (Ryan) discussing other factors relevant to the quality of building and infrastructure:  *“So, build quality is important. There are certain apartment manufacturers, like if it’s Meriton I would strongly advise – I lived in a Meriton for one month and it wasn’t built very well. I’ve inspected some and they’re not very great, so I actually do – I mean, if it’s like a small, medium-sized apartment made by some, I don’t know, a small company, a small building manufacturer, then it doesn’t matter very much, but if it’s a huge thirty-floor one that’s made by one of the big companies like Meriton, or Billbergia, or one of the others, then yeah, because some of those companies like Meriton, I’ve just mentioned, to me I associate it with low build quality. I already know that it’s an apartment that I probably would not live in because of the low build quality, and that can be from bad soundproofing to…the Meriton I lived in for a month, I turned the tap on, and all kinds of noises were coming behind, where the water pipes were, and that kind of stuff.”* (Ryan)  During the interview with participant (Ryan), he talked about his hesitancy to buy a property in Sydney, linking the quality of new builds and off-plan in NSW with the companies responsible for building apartments as well as questionable government processes:  *“I would love high build standards. It would be great, therefore, if the standards were kept to be more accountable. I don’t know about Victoria, where I did live, what it’s like there, but certainly in NSW, yeah, I think there’s been a lot of news stories recently about poor build qualities and that kind of stuff. and things. Yeah, I agree with my friend. I would, you know, I would be very unsure about buying a place in a high-rise apartment block that was built within the last ten to 15 years, just because of the risk…”* (Ryan)  “*So, we’ve seen some recent news stories about the Opal Towers in Sydney and that kind of stuff, places which clearly – I think there’s been some cases, I remember this case where I think an inspector or someone said, “This is the worst case I’ve seen of a building with cracks” and that kind of thing, so you clearly get the impression that something’s not right here when it comes to the building quality of places in NSW*.” (Ryan)  *“Yeah, absolutely, and it’s probably why a friend of mine, and people are hesitant to buy new builds and off the plan builds, is because of the build quality in NSW and what developers can get away with, seemingly. And then when there is an issue, it seems there isn’t really a process of these companies and stuff where there’s minimum liability when there’s a problem, and the cost to rectify these issues falls on to the owners of apartments. Either they have to stump up all of the cost, or in the case of an Opal Towers or something, the building is simply not fit for purpose. You know. The value of those places is going to absolutely plummet...”* (Ryan)  From (Ryan)’s insights into the quality of building structures and apartment buildings to other factors outside the apartment building level, including accountability of build standards in NSW, all the way to election pledges on quality in NSW:  *“And I think when it came to state elections, if one of the parties actually started talking about that and made that a big part of their election pledge, would be to improve that, that is a significant – even though I’m a renter and I don’t have any plans to buy a place in the next few years, to me that would be a significant reason to vote for them. Yeah. I think that’s a big enough issue that affects my voting at a state and Council level.”* (Ryan). It seems quality of apartment buildings sits at the heart of participant (Ryan) and other participants’ insights. |
